# Perceived value of computed tomography imaging for patients with inflammatory bowel disease in the emergency department: a Canadian survey

**DOI:** 10.1093/jcag/gwae001

**Published:** 2024-02-16

**Authors:** Caleb A N Roda, Catherine Dube, Blair D Macdonald, Ian G Stiell, Husein Moloo, Anthony deBuck van Overstraeten, Sanjay Murthy, Ranjeeta Mallick, Jeffrey D McCurdy

**Affiliations:** Department of Medicine, Division of Gastroenterology, The Ottawa Hospital, Ottawa K1Y 1J8, ON, Canada; Department of Medicine, Division of Gastroenterology, The Ottawa Hospital, Ottawa K1Y 1J8, ON, Canada; Department of Medical Imaging, University of Ottawa, Ottawa K1Y 1J8, Canada; The Ottawa Hospital Research Institute, Ottawa K1Y 1J8, Canada; Department of Emergency Medicine, University of Ottawa, Ottawa K1Y 1J8, Canada; Department of Surgery, University of Ottawa, Ottawa K1Y 1J8, Canada; The Ottawa Hospital Research Institute, Ottawa K1Y 1J8, Canada; Department of Surgery, Mount Sinai Hospital, University of Toronto, Toronto M5S 1A8, Canada; Department of Medicine, Division of Gastroenterology, The Ottawa Hospital, Ottawa K1Y 1J8, ON, Canada; The Ottawa Hospital Research Institute, Ottawa K1Y 1J8, Canada; The Ottawa Hospital Research Institute, Ottawa K1Y 1J8, Canada; Department of Medicine, Division of Gastroenterology, The Ottawa Hospital, Ottawa K1Y 1J8, ON, Canada; The Ottawa Hospital Research Institute, Ottawa K1Y 1J8, Canada

**Keywords:** computed tomography imaging, inflammatory bowel disease, emergency department, survey

## Abstract

**Background:**

There are high rates of computed tomography (CT) utilization in the emergency department (ED) for patients with inflammatory bowel disease (IBD), despite guidelines recommending judicious use. We performed a national survey to better understand perceptions and practice patterns of Canadian physicians related to CT imaging in the ED.

**Methods:**

Our survey was developed by a multistep iterative process with input from key stakeholders between 2021 and 2022. It evaluated Canadian gastroenterologists’, surgeons’, and emergency physicians’ (1) perceived rates of IBD findings detected by CT, (2) likelihood of performing CT for specific presentations and (3) comfort in diagnosing IBD phenotypes/complications without CT.

**Results:**

A total of 208 physicians responded to our survey: median age 44 years (IQR, 37–50), 63% male, 68% academic, 44% emergency physicians, 39% gastroenterologists, and 17% surgeons. Compared with emergency physicians and surgeons, gastroenterologists more often perceived that CT would detect inflammation alone and less often IBD complications. Based on established rates in the literature, 13 (16%) gastroenterologists, 33 (40%) emergency physicians, and 21 (60%) surgeons overestimated the rates of at least one IBD complication. Although most physicians were more comfortable diagnosing inflammation compared to IBD complications without CT, gastroenterologists were significantly less likely to recommend CT imaging for non-obstructive/penetrating presentations compared with emergency physicians and surgeons with results that varied by IBD subtype.

**Conclusion:**

This national survey demonstrates differences in physician perceptions and practices regarding CT utilization in the ED and can be used as a framework for educational initiatives regarding appropriate usage of this modality.

## Introduction

Abdominal pelvic computed tomography (APCT) imaging can be an important diagnostic tool in patients with inflammatory bowel disease (IBD). It has the ability to assess areas of the small bowel that are difficult to reach by standard endoscopy and has a high spatial resolution which makes it an accurate tool for detecting complications such as strictures, fistulas, and abscesses. It also has rapid image acquisition time and 24-h availability in most hospitals, which make it an attractive imaging modality for the emergency department (ED).^1,2^

However, potential overuse of APCT in the ED to help diagnose a variety of gastrointestinal signs and symptoms, particularly among patients with IBD, can lead to increased ED wait times and unnecessary radiation exposure, with associated increased risks of malignancy.^3^ This is particularly relevant in patients with IBD where the young age at diagnosis and relapsing pattern may result in repeated visits to the ED and a potential for high cumulative radiation exposure over a lifetime.^4^ Multiple observational studies have estimated high cumulative rates of radiation exposure in patients with IBD, particularly those with Crohn’s disease (CD).^4–9^ As such, multiple consensus guidelines recommend judicious use of APCT imaging in the ED for patients with IBD.^10–17^

Despite the increased awareness of the potential harms of APCT imaging, the rates of APCT utilization have continued to rise over the past two decades among patients with IBD in the ED.^18,19^ Furthermore, only a minority of patients with IBD who undergo APCT imaging have urgent penetrating or structuring complications (abscesses, phlegmon, perforation, or obstruction) suggesting that APCT imaging is not always necessary.^18–26^ To better understand physicians’ preferences and perceived practices regarding APCT utilization in the IBD population, we performed a national survey of gastroenterologists, surgeons, and emergency physicians.

## Methods

We performed a cross-sectional national web-based survey of Canadian gastroenterologists, surgeons, and emergency physicians. The survey was distributed between November 2021 and August 2022 using the SurveyMonkey platform. Participants were contacted directly by publicly available institutional email addresses, and indirectly by the Canadian Association of Gastroenterology, Canadian Association of General Surgeons, and Canadian Society of Colon and Rectal Surgeons newsletters. Duplicate entries were avoided by preventing users with the same IP address access to the survey twice. Ethics approval was granted by the Ottawa Health Science Research Ethics Board through a delegated review process.

### Survey design

Our survey was created using a multistep iterative process with input from specialists in gastroenterology, surgery, emergency medicine, and radiology. The survey underwent two rounds of reviews by each member of our working group (JM, BM, CD, IS, and AD). Prior to survey dissemination, clarity, usability, and technical functionality were tested by multiple physician volunteers. The final survey questions are reported in Table S1.

Our survey consisted of 18 questions: 7 free text or multiple-choice questions for participant demographics; 2 multiple choice questions to assess the perceived likelihood of specific IBD complications (bowel inflammation, bowel obstruction, septic complications [abscess and phlegmon], megacolon and bowel perforation) reported by APCT imaging in the ED; 6 questions using Likert/Likert-type items to assess physician practice patterns pertaining to APCT imaging in the ED (clinical presentations prompting APCT imaging, diagnostic confidence without APCT imaging, and factors influencing the decision to perform APCT imaging for both CD and ulcerative colitis [UC]); and 3 categorical/free text questions assessing the perceived utility of a validated clinical decision support tool and estimated radiation risk of APCT imaging.

### Analysis

For analytic purposes, we converted the data from questions pertaining to the perceived proportion of APCT imaging findings into dichotomous groups using cut-offs based on the average rates of each IBD finding reported in the literature ^6,18–20,23–25,27,28^. Cut-offs included >50% for inflammation alone, ≤25% for bowel obstruction, ≤10% for septic complication, and ≤5% for perforation for CD and ≥75% for inflammation alone, ≤10% for bowel obstruction, ≤10% for septic complication, ≤10% for megacolon, and ≤5% for perforation for UC. Pairwise comparisons between specialties were performed by Chi-squared and Fisher exact statistical tests where appropriate. For Likert/Liker-type questions measuring the likelihood of CT utilization or the likelihood of factors influencing CT utilization, we calculated the mean likelihood with standard deviation for each physician specialty by assigning numerical values to each response (1 for very unlikely, 2 for unlikely, 3 for possible, 4 for likely, and 5 for very likely). Pairwise comparisons between specialties were performed by two-tailed Mann–Whitney *U* testing. Adjustments were not made for multiple comparisons.

## Results

### Participant demographics

A total of 208 physicians responded to our survey. Demographics and specialty type are reported in Table 1. The median age was 44 years (IQR, 37–50) and the majority identified as male (*n* = 132, 63%). Survey participants by specialty included: 92 (44%) emergency physicians, 81 (39%) gastroenterologists, and 35 (17%) surgeons. There was representation from Central provinces (*n* = 133, 64%), Western Canada (*n* = 38, 18%), Prairie provinces, (*n* = 23, 11%), and Atlantic provinces (*n* = 14, 7.0%). There was no representation from the territories. Most participants were from Ontario (*n* = 122, 59%) and practiced at academic centers (*n* = 141, 68%).

**Table 1.** Participant demographics and practice characteristics.

Variables	Gastroenterology (*n* = 81)	Surgery(*n* = 35)	Emergency Medicine (*n* = 92)
**Sex:** *n* (%)			
Male	52 (64)	20 (57)	60 (65)
Female	29 (36)	15 (43)	28 (30)
Non-Binary	0 (0)	0 (0)	1 (1.1)
Other	0 (0)	0 (0)	3 (3.3)
**Age**: median (IQR)	45 (14)	44 (10)	42 (15)
**Age**: mean (STD)	46 (9)	47 (8)	43 (10)
**Province or territory of practice:** *n* (%)			
Western Canada^a^	8 (4.0)	2 (1.0)	28 (13)
Prairie Provinces^b^	18 (8.6)	4 (8.7)	1 (0.5)
Central Canada and Quebec^c^	42 (20)	28 (13)	63 (30)
Atlantic Region^d^	13 (6.3)	1 (0.5)	0 (0)
Territories^e^	0 (0)	0 (0)	0 (0)
**Years in practice**: median (IQR)	12 (7–17)	12 (5–16)	10 (35–50)
**Practice environment:** *n* (%)			
Academic	49 (61)	22 (63)	70 (76)
Community	31 (38)	12 (34)	19 (21)
Other	1 (1.2)	1 (2.9)	3 (3.3)
**Percentage of IBD patients in practice:** median (IQR)	40 (20–63)	7 (5–15)	2 (1–5)

^a^Western Canada = British Columbia.

^b^Prairie Provinces = Alberta, Saskatchewan, and Manitoba.

^c^Central Provinces = Ontario and Quebec.

^d^Atlantic Provinces = New Brunswick, Nova Scotia, Newfoundland, Prince Edward Island.

^e^Territories = Yukon, Northwest Territories, and Nunavut.

### Perceived proportion of APCT imaging with specific findings

Participants were asked to estimate the prevalence of specific APCT findings in the ED among IBD patients. In UC, gastroenterologists were more likely to report higher perceived rates of inflammation alone and lower perceived rates of IBD complications (Figure S1a and Table 2). Based on established rates of APCT findings from the literature for patients with UC, gastroenterologists were significantly less likely to underestimate the proportion of APCT imaging with inflammation alone compared with surgeons (64% vs 77%; *P* = 0.211) and emergency physicians (64% vs 82%; *P* = 0.011). Gastroenterologists were also significantly less likely to overestimate the proportion of scans with at least one complication compared with surgeons (16% vs 60%; *P* < 0.001) and emergency physicians (16% vs 40%; *P* < 0.001). A proportion of gastroenterologists, surgeons, and emergency physicians overestimated the proportion of scans with bowel obstruction (4% vs 26% vs 20%), septic complications (4% vs 26% vs 15% for), mega colon (15% vs 43% vs 14%), and bowel perforation (5% vs 37% vs 26%), respectively.

**Table 2.** Perceived proportion of specific findings on abdominal pelvic computed tomography imaging for patients with ulcerative colitis and Crohn’s disease in the emergency department stratified by physician speciality.

Proportion of positive findings	GI	*N* (%) Surg	EM	Chi-squared (*P* values)		
				GI versus Surg	GI versus EM	Surg versus EM
**Ulcerative colitis**						
Inflammation alone (>75%)	28 (36)	7 (23)	18 (18)	0.211	0.011	0.499
Bowel obstruction (≤10%)	77 (96)	25 (74)	61 (80)	<0.001	0.002	0.429
Septic complication (≤10%)^a^	78 (96)	26 (74)	68 (85)	0.001	0.012	0.171
Megacolon (≤10%)	69 (85)	20 (57)	67 (86)	0.001	0.898	<0.001
Bowel perforation (≤5%)	77 (95)	22 (63)	57 (74)	<0.001	<0.001	0.229
**Crohn’s disease**						
Inflammation alone (>50%)	39 (49)	8 (24)	36 (46)	0.012	0.744	0.024
Bowel obstruction (≤25%)	59 (74)	18 (51)	70 (86)	0.019	0.044	<0.001
Septic complication (≤10%)^a^	45 (56)	8 (23)	64 (80)	<0.001	<0.001	<0.001
Bowel perforation (≤5%)	75 (94)	11 (31)	51 (66)	<0.001	<0.001	<0.001

^a^Abscess or phlegmon.

Abbreviations: EM, emergency medicine; GI, gastroenterology; Surg, surgery.

In CD, gastroenterologists and emergency physicians reported higher perceived rates of inflammation alone and lower rates of most IBD complications compared with surgeons (Figure S1b and Table 2). Based on established rates of APCT findings from the literature for patients with CD, gastroenterologists and emergency physicians were significantly less likely to underestimate the proportion of APCT imaging with inflammation alone compared to surgeons (51% vs 76%; *P* = 0.012 and 54% vs 76%; *P* = 0.024), respectively. Gastroenterologists and emergency physicians were also significantly less likely to overestimate the proportion of scans with at least one complication compared to surgeons (49% vs 89%; *P* < 0.001 and 40% vs 89%; *P* < 0.001), respectively. A proportion of gastroenterologists, surgeons, and emergency physicians overestimated the proportion of scans with bowel obstruction (26% vs 49% vs 14%), septic complications (44% vs 77% vs 20%), and bowel perforation (6% vs 69% vs 34%), respectively.

### Clinical presentations prompting APCT imaging

**Figure 1.**
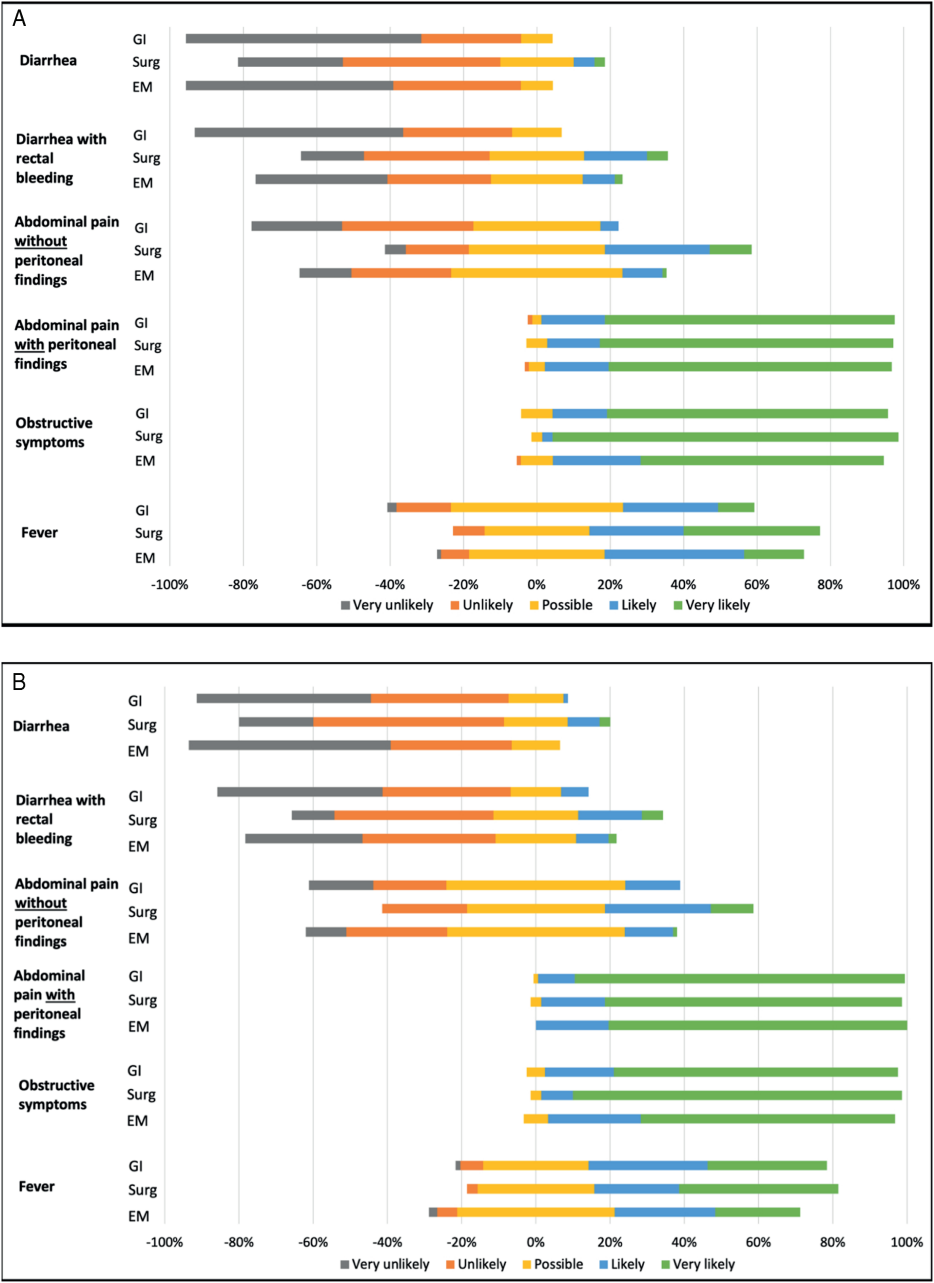
Likelihood of recommending APCT imaging in the emergency department for patients with (A) ulcerative colitis and (B) Crohn’s disease based on various clinical presentations stratified by physician specialty: gastroenterology (GI), surgery (Surg), and emergency medicine (EM).

Participants were asked how likely they would recommend APCT imaging in the ED based on various clinical presentations (Figure 1 and Table S2). Physicians from each specialty were most likely to recommend APCT imaging for abdominal pain with peritoneal findings and obstructive symptoms. In UC, gastroenterologists were less likely to recommend APCT imaging for diarrhoea (mean likelihood [SD]; 1.44 [0.65] vs 2.11 [0.99]; *P* < 0.001), rectal bleeding (1.57 [0.72] vs 2.60 [1.14]; *P* < 0.001), and abdominal pain without peritoneal findings (2.20 [0.87] vs 3.22 [1.05]; *P* < 0.001) compared with surgeons. Similarly, emergency physicians were less likely to recommend APCT imaging for diarrhoea (1.52 [0.65] vs 2.11 [0.99]; *P* = 0.002), rectal bleeding (2.13 [1.07] vs 2.60 [1.14]; *P* = 0.04) and abdominal pain without peritoneal findings (2.58 [0.90] vs. 3.22 [1.05]; *P* = 0.003) compared with surgeons. There were no major differences in practice patterns between gastroenterologists and emergency physicians. Similar trends were observed in CD.

### Level of comfort in clinical diagnosis without APCT imaging

**Figure 2.**
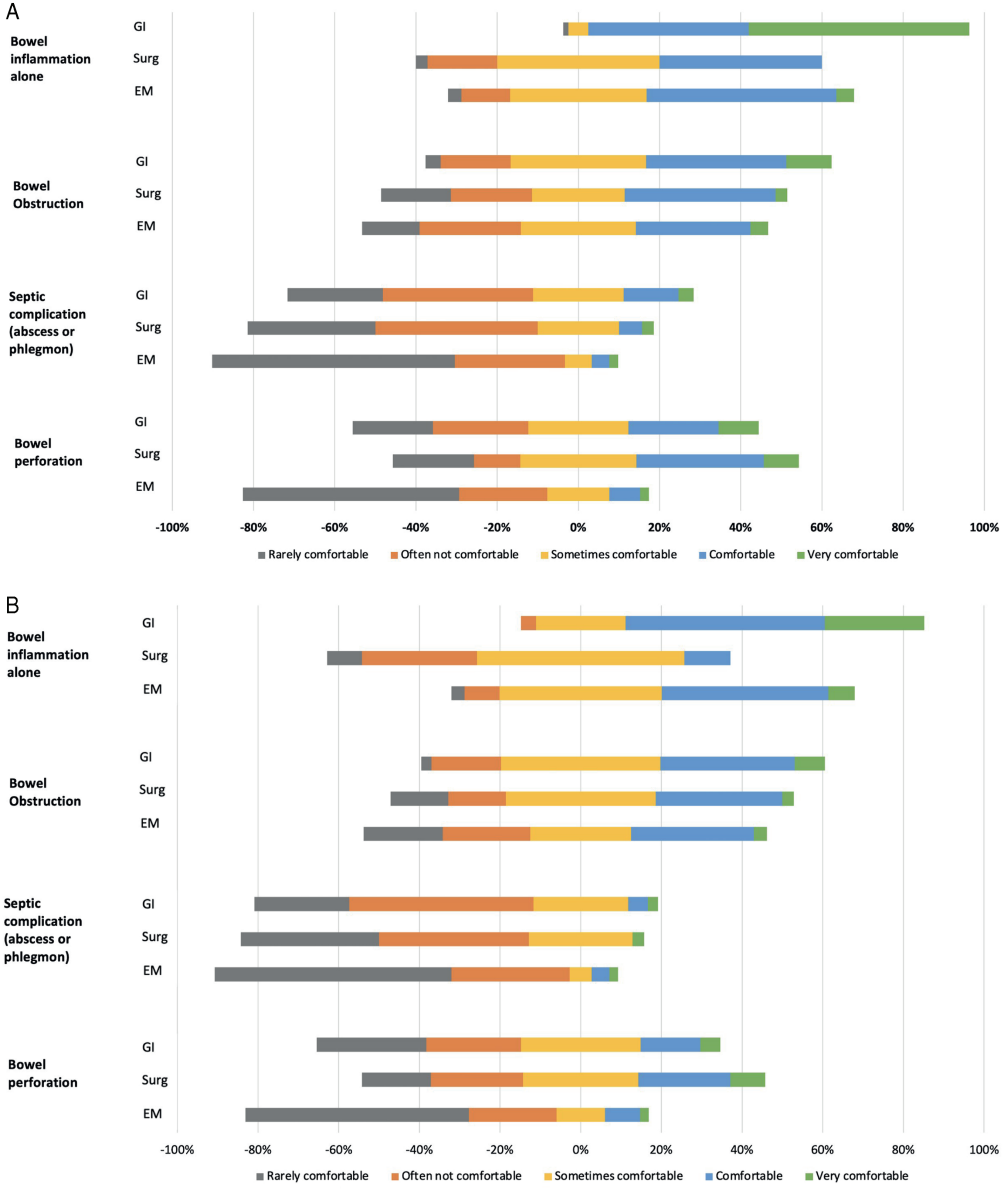
Level of comfort in diagnosing various disease phenotypes/complications without APCT imaging in the emergency department for (A) ulcerative colitis and (B) Crohn’s disease stratified by physician speciality: gastroenterology (GI), surgery (Surg), and emergency medicine (EM).

Participants were asked about their level of comfort in diagnosing specific disease phenotypes/complications without APCT imaging (Figure 2 and Table S3). Similar trends were observed in UC and CD. In general, physicians from each specialty were more comfortable in diagnosing inflammation and less comfortable diagnosing septic complications without APCT imaging. In UC, gastroenterologists reported being more comfortable diagnosing inflammation without APCT imaging compared with surgeons (mean comfort [SD]; 4.46 [0.71] vs 3.17 [0.82]; *P* < 0.001) and emergency physicians (4.46 [0.71] vs 3.37 [0.87]; <0.001). Both gastroenterologists and surgeons reported being more comfortable diagnosing septic complications compared with emergency physicians, respectively (2.37 [1.10] vs 1.62 [0.95]; *P* < 0.001 and 2.09 [1.01] vs 1.62 [0.95]; *P* = 0.009).

For CD, gastroenterologists reported being more comfortable diagnosing inflammation without APCT imaging compared with surgeons (3.95 [0.79] vs 2.66 [0.80]; *P* < 0.001) and emergency physicians (3.95 [0.79] vs 3.39 [0.86]; *P* < 0.001), respectively. Gastroenterologists reported being more comfortable in diagnosing bowel obstruction without APCT imaging compared with emergency physicians (3.26 [0.92] vs 2.76 [1.18]; *P* = 0.011). Both gastroenterologists and surgeons were more comfortable diagnosing septic complications (2.17 [0.93] vs 1.62 [0.94]; *P* < 0.001 and 2.00 [0.94] vs 1.62 [0.94]; *P* = 0.019) and perforations (2.47 [1.18] vs 1.80 [1.09]; *P* < 0.001 and 2.83 [1.22] vs 1.80 [1.09]; *P* < 0.001) without APCT imaging compared with emergency physicians, respectively. Otherwise, there were no significant differences in the reported level of comfort in diagnosing obstructions, septic complications, or perforations between gastroenterologists and surgeons.

### Factors influencing the decision to perform APCT imaging

Participants were asked how likely various factors influenced their decision to perform APCT imaging in the ED (Figure S2 and Table S4). The results were broadly similar for UC and CD and between specialties. In general, physicians from each specialty were more likely to perform APCT imaging to rule out IBD complications and non IBD aetiologies and less likely to perform APCT imaging because of patients’ requests or to minimize the risk of litigation. Of note, for patients with UC, gastroenterologists reported being less likely to perform APCT imaging because the clinical history/physical exam and laboratory testing/x-ray are not reliable enough compared with surgeons (mean likelihood [SD]; 2.74 [1.08] vs 3.37 [0.94]; *P* = 0.005). Similarly, gastroenterologists reported being less likely to perform APCT imaging to rule out complications of UC compared with surgeons (3.10 [0.90] vs 3.83 [0.86]; *P* < 0.001) and emergency physicians (3.10 [0.90] vs 3.07 [0.97]; *P* < 0.001), respectively. For CD, emergency physicians reported being less likely to perform APCT imaging because the clinical history/physical exam and laboratory testing/x-ray are not reliable enough compared with surgeons (3.17 [0.93] vs 3.69 [0.72]; *P* = 0.008). Finally, for both UC and CD, emergency physicians reported being more likely to performing APCT imaging for patients because the consultants would want it compared with gastroenterologists (2.38 [1.08] vs 3.85 [1.00]; *P* < 0.001 for UC and 2.79 [1.20] vs 3.85 [1.06]; <0.001 for CD) and surgeons (2.69 [1.18] vs 3.85 [1.06]; *P* < 0.001 for UC and 2.69 [1.23] vs 3.85 [1.06]; <0.001 for CD).

## Discussion

The findings of this national survey provide valuable insights into physician perceptions and factors contributing to APCT utilization in the ED for patients with IBD. We found significant variation in the perceived prevalence of IBD-related findings on APCT imaging and practice patterns between physician specialties. Overestimation of IBD complications, performing APCT imaging for non-obstructive/penetrating indications, and a lack of confidence in the reliability of the clinical history, physical exam, and basic investigations were determined to influence the use of APCT imaging in the ED for patients with IBD and may be reasons for the high rates of APCT utilization.

One key finding of our study is the variation in the perceived rates of IBD-related findings among different physician specialties. Gastroenterologists reported higher perceived rates of inflammation alone and lower rates of IBD complications compared with surgeons and emergency physicians. This discrepancy in perception may be attributed to referral bias, as surgeons are more likely to encounter patients with IBD complications, leading to a higher proportion of their patients having such complications. Additionally, the variation may be influenced by volume of IBD exposure and experience with IBD-related complications.

Importantly, a proportion of physicians overestimated the rates of IBD complications, particularly in UC where IBD complications are rare.^6,18,20,26,27^ Most published studies report rates of obstructive and penetrating complications of less than 5% each on APCT imaging in the ED.^6,20,26,27^ These rates are much lower than the perceived rates reported by emergency physicians and surgeons in our study. In CD, disease complications detected by APCT imaging are more common,^6,18,19,22–26,28^ with most published series reporting rates of obstructive complications of 10–27%, 7–10% for septic complications, but less than 5% for penetrating complications.^6,18,19,23–25,27,28^ Interestingly, 32% of gastroenterologists and 55% of emergency physicians in our study underestimated the proportion of imaging studies with obstructive complications, while 44% of gastroenterologists and 77% of surgeons overestimated the proportion of imaging studies with septic complications and 69% of surgeons and 34% of emergency physicians overestimated the proportion of imaging studies with perforation.

Another important finding of our study is related to the use of APCT imaging for non-obstructive/penetrating clinical presentations. A proportion of physicians still indicated at least a possibility of performing APCT imaging for indications such as diarrhoea alone, rectal bleeding, and abdominal pain without peritoneal findings. This suggests a need for education and awareness among physicians regarding the appropriateness of APCT imaging for these presentations. Although several guidelines exist for APCT imaging in the ED for patients with IBD^10–17^ they were developed primarily by gastroenterologists and radiologists and may not address the specific practice needs of surgeons and emergency physicians. As a result, multidisciplinary guidelines are required to ensure consistent and evidence-based decision-making.

In general, all physician specialties were more comfortable diagnosing inflammation alone compared with IBD complications. However, a substantial proportion of physicians indicated that they did not feel the clinical history, physical exam, and basic investigations such as laboratory findings and abdominal x-ray to be reliable enough. This highlights the need for predictive tools to enhance physicians’ confidence in selecting patients at highest risk of IBD-related complications to allow for a more targeted approach to APCT imaging. Although a number of clinical prediction tools have been developed for IBD-related complications, none have been prospectively validated.^23,25,26,29,30^ This is important since our study found that 73 (90%) gastroenterologists, 88 (96%) emergency physicians, and 28 (80%) surgeons expressed a willingness to use a validated clinical decision support tool if available.

Collectively, these findings provide insights into why the rates of APCT utilization remain so high for patients with IBD, despite the potential harmful impacts of ionizing radiation, and the importance of reducing wait times for diagnostic imaging and associated healthcare costs. Furthermore, there has also been greater awareness of the global environmental impacts of radiologic procedures, including ACPT scans, and the importance of reducing unnecessary energy consumption and associated waste materials.^31–34^ In keeping with these themes, recent Choosing Wisely guidelines^15^ and expert consensus statements^10–12,14,16^ recommending judicious use of APCT imaging in the ED and reductions in APCT utilization in the general population presenting to the ED with abdominal pain.^35^ However, since these guidelines, a recent study from Ottawa, Canada, demonstrated a continued annual increase of 2.7% in APCT utilization in IBD between 2009 and 2018.^18^

To address the findings of our study, future work should focusing on (1) Physician education on the expected rates of IBD complications and clinical scenarios for which APCT is unnecessary as a baseline investigation; (2) development of multidisciplinary guidelines outlining the appropriateness of APCT imaging for specific clinical presentations; (3) validation of clinical predictive tools to identify patients at highest risk of IBD complications; and (4) increasing access to point-of-care faecal biomarkers to assess for inflammation as well as alternate imaging modalities, such as magnetic resonance imaging and intestinal ultrasound, that do not rely on ionizing radiation.

To our knowledge, this is the first national survey focused on clinician perceptions and preferences of APCT imaging in the ED for patients with IBD. The strengths of our study include the collaborative design with input from key stakeholders in multiple specialties, representation from the majority of regions across Canada and an analysis that was stratified by physician specialty. Our study also contained limitations. Most notably our survey response rates were low. Given that our survey was distributed, in part, by division heads and societal distribution lists, we were not able to calculate a response rate. However, as is the case for most surveys, our response rate was low. Furthermore, the small numbers of respondents from some subgroups prevented us from doing additional subgroup analysis and may limit the generalizability of our findings. Additionally, our survey was based on physician’s perceptions instead of a practice audit. As a result, our study design is subject to recall bias and may not reflect the true practice patterns of physicians. It is also important to acknowledge that while there were statistically significant differences in practice patterns between specialties, the effect sizes for some comparisons were small and may not be clinically meaningful. Finally, our survey questions may not have captured the full breadth of physicians’ perceptions and practices regarding APCT use in the ED and do not address other potential contributors to APCT use, such as physician and nurse staffing in the ED, consultation rates with subspecialists prior to ordering APCT, access to APCT in the ED, and access to alternative modalities such as U/S and MRI.

In conclusion, our national survey on physician perception and preferences of APCT imaging in the ED for patients with IBD identified several factors that may explain the overuse of APCT imaging and helps lay an initial framework to guide educational initiatives on APCT imaging in the ED for patients with IBD. Our hope is that this and future work will provide a more rational approach to guide the appropriateness of APCT imaging in the ED for patients with IBD and will ultimately help reduce the cumulative rates of ionizing radiation for our patients with IBD, burden on healthcare resources, and global carbon footprint.

## Supplementary data

Supplementary data are available at *Journal of the Canadian Association of Gastroenterology* online.

gwae001_suppl_Supplementary_Tables_1-4_Figures_1-2

gwae001_suppl_Supplementary_Material

## Data Availability

The data that support the findings of this study are available on request from the corresponding author (Jeffrey D. McCurdy). The data are not publicly available due to their containing information that could compromise the privacy of research participants.
