# Supplementary material for: Perceived value of computed tomography imaging for patients with inflammatory bowel disease in the emergency department: a Canadian survey
Source: J Can Assoc Gastroenterol. 2024 Feb 16;7(3):261–8. doi: 10.1093/jcag/gwae001 (PMC11149658; doi:10.1093/jcag/gwae001)
Supplement: gwae001_suppl_Supplementary_Tables_1-4_Figures_1-2 [file gwae001_suppl_supplementary_tables_1-4_figures_1-2.zip › gwae001/Supplemental Table and Figures.docx]

| **Supplementary Table 1.** Survey questions | | | | | | | | | | | | | | | | | | | | | | |  |  |
| --- | --- | --- | --- | --- | --- | --- | --- | --- | --- | --- | --- | --- | --- | --- | --- | --- | --- | --- | --- | --- | --- | --- | --- | --- |
| ***Q1. What is your medical speciality?*** | | | | | | | | | | | | | | | | | | | | | | |  |  |
| Emergency medicine | |  | | | | | |  | | | | |  | | | |  |  | | | | |  |  |
| Gastroenterology | |  | | | | | |  | | | | |  | | | |  |  | | | | |  |  |
| General Surgery | |  | | | | | |  | | | | |  | | | |  |  | | | | |  |  |
| ***Q2. Based on the following clinical presentation, how often would you recommend abdominal CT imaging for patients with ulcerative colitis in the ED?*** | | | | | | | | | | | | | | | | | | | | | | |  |  |
|  | Very unlikely | | | | | | | Unlikely | | | | | Possible | | | | Likely | Very likely | | | | |  |  |
| Diarrhea |  | | | | | | |  | | | | |  | | | |  |  | | | | |  |  |
| Diarrhea with rectal bleeding |  | | | | | | |  | | | | |  | | | |  |  | | | | |  |  |
| Abdominal pain without peritoneal findings |  | | | | | | |  | | | | |  | | | |  |  | | | | |  |  |
| Abdominal pain with peritoneal findings |  | | | | | | |  | | | | |  | | | |  |  | | | | |  |  |
| Obstructive symptoms |  | | | | | | |  | | | | |  | | | |  |  | | | | |  |  |
| Fever |  | | | | | | |  | | | | |  | | | |  |  | | | | |  |  |
| ***Q3. Based on the following clinical presentation, how often would you recommend abdominal CT imaging for patients with Crohn’s disease in the ED?*** | | | | | | | | | | | | | | | | | | | | | | |  |  |
|  | Very unlikely | | | | | | | Unlikely | | | | | Possible | | | | Likely | Very likely | | | | |  |  |
| Diarrhea |  | | | | | | |  | | | | |  | | | |  |  | | | | |  |  |
| Diarrhea with rectal bleeding |  | | | | | | |  | | | | |  | | | |  |  | | | | |  |  |
| Abdominal pain without peritoneal findings |  | | | | | | |  | | | | |  | | | |  |  | | | | |  |  |
| Abdominal pain with peritoneal findings |  | | | | | | |  | | | | |  | | | |  |  | | | | |  |  |
| Obstructive symptoms |  | | | | | | |  | | | | |  | | | |  |  | | | | |  |  |
| Fever |  | | | | | | |  | | | | |  | | | |  |  | | | | |  |  |
| ***Q4. How common are the following CT findings in patients with ulcerative colitis who present to the ED with gastrointestinal symptoms?*** | | | | | | | | | | | | | | | | | | | | | | |  |  |
|  | | | | | 0-5% | | 6-10% | | 11-25% | | | | 26-50% | | | | 51-75% | >75% | | Unsure | | |  |  |
| Inflammation alone |  | | | | | |  | | | | |  | | |  | |  |  | |  | | |  |  |
| Bowel obstruction |  | | | | | |  | | | | |  | | |  | |  |  | |  | | |  |  |
| Septic complication (abscess or phlegmon) |  | | | | | |  | | | | |  | | |  | |  |  | |  | | |  |  |
| Megacolon or distended colon |  | | | | | |  | | | | |  | | |  | |  |  | |  | | |  |  |
| Bowel perforation |  | | | | | |  | | | | |  | | |  | |  |  | |  | | |  |  |
| ***Q5. How common are the following CT findings in patients with Crohn’s disease who present to the ED with gastrointestinal symptoms?*** | | | | | | | | | | | | | | | | | | | | | | |  |  |
|  | | | | | 0-5% | | 6-10% | | | 11-25% | | | 26-50% | | | | 51-75% | >75% | | Unsure | | |  |  |
| Inflammation alone | | |  | | |  | | | | | |  | |  | | |  | |  | |  | |  |  |
| Bowel obstruction | | |  | | |  | | | | | |  | |  | | |  | |  | |  | |  |  |
| Septic complication (abscess or phlegmon) | | |  | | |  | | | | | |  | |  | | |  | |  | |  | |  |  |
| Bowel perforation | | |  | | |  | | | | | |  | |  | | |  | |  | |  | |  |  |
| ***Q6. How comfortable are you in diagnosing the following based on clinical history, physical exam, labs and x-ray without an abdominal CT scan for patients with ulcerative colitis?*** | | | | | | | | | | | | | | | | | | | | | | |  |  |
|  | Rarely uncomfortable | | | | | | Often not comfortable | | | | | Sometimes comfortable | | | | Comfortable | | Very Comfortable | | | | |  |  |
| Bowel inflammation |  | | | | | | |  | | | | |  | | | |  |  | | | | |  |  |
| Bowel obstruction |  | | | | | | |  | | | | |  | | | |  |  | | | | |  |  |
| Septic complication (abscess or phlegmon) |  | | | | | | |  | | | | |  | | | |  |  | | | | |  |  |
| Bowel perforation |  | | | | | | |  | | | | |  | | | |  |  | | | | |  |  |
| ***Q7. How comfortable are you in diagnosing the following based on clinical history, physical exam, labs and x-ray without an abdominal CT scan for patients with Crohn’s disease?*** | | | | | | | | | | | | | | | | | | | | | | |  |  |
|  | Rarely uncomfortable | | | | | | Often not comfortable | | | | | Sometimes comfortable | | | Comfortable | | | Very Comfortable | | | | |  |  |
| Bowel inflammation |  | | | | | | |  | | | | |  | | | |  |  | | | | |  |  |
| Bowel obstruction |  |  |  |  |  |  |  |  |  |  |  |  |  |  |  |  |  |  |  |  |  |  |  |  |
| Septic complication (abscess or phlegmon) |  | | | | | | |  | | | | |  | | | |  |  | | | | |  |  |
| Bowel perforation |  | | | | | | |  | | | | |  | | | |  |  | | | | |  |  |
| ***Q8. How much do the following reasons influence your decision to recommend abdominal CT imaging for patients with ulcerative colitis who present to the ED?*** | | | | | | | | | | | | | | | | | | | | | | |  |  |
|  | | | | | Very unlikely | | | | | | Unlikely | | Possible | | | | Likely | Very likely | | | | |  |  |
| The clinical history, physical exam, labs and x-ray findings are not reliable enough to determine the cause of symptoms | | | | |  | | |  | | | | |  | | | |  |  | | | | |  |  |
| Rule out a acute complication of ulcerative colitis disease | | | | |  | | |  | | | | |  | | | |  |  | | | | |  |  |
| Evaluate for an alternative cause when a non-IBD etiology is suspected | | | | |  | | |  | | | | |  | | | |  |  | | | | |  |  |
| Patients request it  Minimize the risk of litigation from missing a clinically important finding | | | | |  | | |  | | | | |  | | | |  |  | | | | |  |  |
| Consultant would want or request a CT scan prior to consultation | | | | |  | | |  | | | | |  | | | |  |  | | | | |  |  |
| Other reason (please specify) | | | | |  | | |  | | | | |  | | | |  |  | | | | |  |  |
| ***Q9. How much do the following reasons influence your decision to recommend abdominal CT imaging for patients with Crohn’s disease who present to the ED?*** | | | | | | | | | | | | | | | | | | | | | | |  |  |
|  | | | | Very unlikely | | | | | | Unlikely | | | Possible | | | | Likely | Very likely | | | | |  |  |
| The clinical history, physical exam, labs and x-ray findings are not reliable enough to determine the cause of symptoms | | | |  | | | | | |  | | |  | | | |  |  | | | | |  |  |
| Rule out a acute complication of Crohn’s disease | | | |  | | | | | |  | | |  | | | |  |  | | | | |  |  |
| Evaluate for an alternative cause when a non-IBD etiology is suspected | | | |  | | | | | |  | | |  | | | |  |  | | | | |  |  |
| Patients request it  Minimize the risk of litigation from missing a clinically important finding | | | |  | | | | | |  | | |  | | | |  |  | | | | |  |  |
| Consultant would want or request a CT scan prior to consultation | | | |  | | | | | |  | | |  | | | |  |  | | | | |  |  |
| Other reason (please specify) | | | |  | | | | | |  | | |  | | | |  |  | | | | |  |  |
| ***Q10. If available, would you use a validated clinical decision support tool to guide when to recommend abdominal CT imaging for patients with ulcerative colitis?***  ***Leave question blank if unsure.*** | | | | | | | | | | | | | | | | | | | | | |  |  |  |
| Yes  No | | | | | | | | | | | | | | | | | | | | | |  |  |  |
| ***Q11. If available, would you use a validated clinical decision support tool to guide when to recommend abdominal CT imaging for patients with Crohn’s disease?***  ***Leave question blank if unsure.*** | | | | | | | | | | | | | | | | | | | | | |  |  |  |
| Yes  No | | | | | | | | | | | | | | | | | | | | | |  |  |  |
| ***Q12.According to the average radiation dose of an abdominal CT scan, how many scans are required to place an individual at increased risk of complications from excess radiation exposure?*** | | | | | | | | | | | | | | | | | | | | | |  |  |  |
| Free Text | | | | | | | | | | | | | | | | | | | | | |  |  |  |
| ***Q13.* What is your age?** | | | | | | | | | | | | | | | | | | | | | |  |  |  |
| Free Text | | | | | | | | | | | | | | | | | | | | | |  |  |  |
| ***Q14. What is your sex?***  Male  Female  Non-binary  Other | | | | | | | | | | | | | | | | | | | | | |  |  |  |
| ***Q15. How many years have you been practicing independently?***  Free Text | | | | | | | | | | | | | | | | | | | | | |  |  |  |
| ***Q16. Which province or territory do you practice in?*** | | | | | | | | | | | | | | | | | | | | | |  |  |  |
| British Columbia | | | | | | | | | | | | | | | | | | | | | |  |  |  |
| Alberta | | | | | | | | | | | | | | | | | | | | | |  |  |  |
| Saskatchewan | | | | | | | | | | | | | | | | | | | | | |  |  |  |
| Manitoba | | | | | | | | | | | | | | | | | | | | | |  |  |  |
| Ontario | | | | | | | | | | | | | | | | | | | | | |  |  |  |
| Quebec | | | | | | | | | | | | | | | | | | | | | |  |  |  |
| New Brunswick | | | | | | | | | | | | | | | | | | | | | |  |  |  |
| Nova Scotia | | | | | | | | | | | | | | | | | | | | | |  |  |  |
| Newfoundland | | | | | | | | | | | | | | | | | | | | | |  |  |  |
| Prince Edward Island | | | | | | | | | | | | | | | | | | | | | |  |  |  |
| Yukon | | | | | | | | | | | | | | | | | | | | | |  |  |  |
| Northwest Territories | | | | | | | | | | | | | | | | | | | | | |  |  |  |
| Nunavut | | | | | | | | | | | | | | | | | | | | | |  |  |  |
| ***17. Which best describes your practice environment?*** | | | | | | | | | | | | | | | | | | | | | |  |  |  |
| Academic practice | | | | | | | | | | | | | | | | | | | | | |  |  |  |
| Community practice | | | | | | | | | | | | | | | | | | | | | |  |  |  |
| Other (please specify) | | | | | | | | | | | | | | | | | | | | | |  |  |  |
| ***18. What percentage of patients in your practice (GI, surgery, or emergency medicine) have IBD?*** | | | | | | | | | | | | | | | | | | | | | |  |  |  |

Free Text

| **Supplementary Table 2.** Likelihood of recommending APCT imaging in the emergency department for patients with ulcerative colitis and Crohn’s disease based on various clinical presentations stratified by physician specialty. | | | | | | | | | | | | |
| --- | --- | --- | --- | --- | --- | --- | --- | --- | --- | --- | --- | --- |
| **Dominant clinical presentation** | **GI** | **Mean (SD)**  **Surg** | **Mann-Whitney U test (p value)**  **EM GI vs Surg GI vs EM Surg vs EM** | | | | | | | | | |
| **Ulcerative colitis** |  |  |  | |  | |  | | | |  | |
| Diarrhea | 1.44 (0.65) | 2.11 (0.99) | 1.52 (0.65) | | <0.001 | | | | 0.796 | | | 0.002 |
| Rectal bleeding | 1.57 (0.72) | 2.60 (1.14) | 2.13 (1.07) | | <0.001 | | | | <0.001 | | | 0.044 |
| Abdominal pain | 2.20 (0.87) | 3.22 (1.05) | 2.58 (0.90) | | <0.001 | | | | 0.010 | | | 0.003 |
| Peritoneal findings | 4.74 (0.59) | 4.74 (0.56) | 4.71 (0.60) | | 0.960 | | | | 0.810 | | | 0.818 |
| Obstructive symptom | 4.68 (0.62) | 3.91 (1.01) | 4.55 (0.70) | | 0.136 | | | | 0.308 | | | 0.016 |
| Fever | 3.26 (0.92) | 4.06 (0.42) | 3.61 (0.89) | | 0.003 | | | | 0.016 | | | 0.121 |
| **Crohn’s disease** |  | | |  | | | | | | | | |
| Diarrhea | 1.70 (0.77) | 2.23 (0.97) | 1.59 (0.71) | | | 0.009 | | | | 0.363 | | <0.001 |
| Rectal bleeding | 1.84 (0.93) | 2.63 (1.09) | 2.14 (1.03) | | | <0.001 | | | | 0.059 | | 0.029 |
| Abdominal pain | 2.60 (0.94) | 3.29 (0.96) | 2.66 (0.88) | | | 0.004 | | | | 0.873 | | 0.005 |
| Peritoneal findings | 4.88 (0.37) | 4.77 (0.49) | 4.80 (0.40) | | | 0.447 | | | | 0.352 | | 0.936 |
| Obstructive symptom | 4.72 (0.55) | 4.86 (0.43) | 4.62 (0.61) | | | 0.313 | | | | 0.363 | | 0.084 |
| Fever | 3.88 (0.98) | 4.06 (0.94) | 3.63 (0.97) | | | 0.418 | | 0.969 | | | | 0.042 |
| EM, emergency medicine; GI, gastroenterology; Surg, surge | | | | | | | | | | | | |

| **Supplementary Table 3.** Level of comfort in diagnosing various disease phenotypes/complications without APCT imaging for patients with ulcerative colitis and Crohn’s disease in the emergency department stratified by physician speciality | | | | | | | | | | |
| --- | --- | --- | --- | --- | --- | --- | --- | --- | --- | --- |
| **Complications of IBD** | | **Mean (SD)**  **GI Surg EM** | | | | | **Man-Whitney U Test (p value)**  **GI vs Surg GI vs EM Surg vs EM** | | | |
| **Ulcerative colitis** | |  |  | |  | |  | |  |  |
| Inflammation | 4.46 (0.71) | | | 3.17 (0.82) | | 3.37 (0.87) | | <0.001 | <0.001 | 0.250 |
| Obstruction | 3.32 (1.01) | | | 2.88 (1.18) | | 2.84 (1.12) | | 0.114 | 0.008 | 0.757 |
| Septic complication† | 2.37 (1.10) | | | 2.09 (1.01) | | 1.62 (0.95) | | 0.215 | <0.001 | 0.009 |
| Bowel perforation | 2.79 (1.27) | | | 2.97 (1.27) | | 1.84 (1.08) | | 0.453 | <0.001 | <0.001 |
| **Crohn’s disease** |  | | | | | | |  | | |
| Inflammation | 3.95 (0.79) | | | 2.66 (0.80) | | 3.39 (0.86) | | <0.001 | <0.001 | <0.001 |
| Bowel Obstruction | 3.26 (0.92) | | | 2.94 (1.08) | | 2.76 (1.18) | | 0.238 | 0.011 | 0.472 |
| Septic complication† | 2.17 (0.93) | | | 2.00 (0.94) | | 1.62 (0.94) | | 0.379 | <0.001 | 0.019 |
| Bowel perforation | 2.47 (1.18) | | | 2.83 (1.22) | | 1.80 (1.09) | | 0.158 | <0.001 | <0.001 |
| †Abscess or phlegmon  EM, emergency medicine; GI, gastroenterology; Surg, surgery | | | | | | | | | | |

| **Supplementary Table 4.** Factors influencing the decision to perform APCT imaging for patients with ulcerative colitis and Crohn’s disease stratified by physician specialty | | | | | | |
| --- | --- | --- | --- | --- | --- | --- |
| **Influencing Factors** | **Mean (SD)**  **GI Surg EM** | | | **Mann-Whitney U Test (p value)**  **GI vs Surg GI vs EM Surg vs EM** | | |
| **Ulcerative colitis** |  |  |  |  |  |  |
| The clinical history, physical exam, labs and X-ray findings are not reliable enough | 2.74 (1.08) | 3.37 (0.94) | 3.07 (0.97) | 0.005 | 0.061 | 0.107 |
| Rule out an acute complication of ulcerative colitis | 3.10 (0.90) | 3.83 (0.86) | 3.66 (0.89) | <0.001 | <0.001 | 0.472 |
| Evaluate for alternative causes when a non-IBD etiology is suspected | 3.44 (0.87) | 3.69 (0.87) | 3.55 (0.89) | 0.267 | 0.542 | 0.529 |
| Patients request it | 1.88 (0.94) | 1.91 (0.89) | 2.22 (0.96) | 0.787 | 0.021 | 0.101 |
| Minimize the risk of litigation from missing a clinically important finding | 2.25 (0.93) | 2.29 (1.07) | 2.40 (1.08) | 0.984 | 0.368 | 0.522 |
| Consultant would want or request a CT scan prior to consultation | 2.38 (1.08) | 2.69 (1.18) | 3.85 (1.00) | 0.250 | <0.001 | <0.001 |
| **Crohn’s disease** |  |  |  |  |  |  |
| The clinical history, physical exam, labs and X-ray findings are not reliable | 3.47 (0.85) | 3.69 (0.72) | 3.17 (0.93) | 0.201 | 0.067 | 0.008 |
| Rule out an acute complication of Crohn’s disease | 4.01 (0.73) | 4.23 (0.55) | 3.92 (0.84) | 0.204 | 0.624 | 0.110 |
| Evaluate for alternative causes when a non-IBD etiology is suspected | 3.64 (0.84) | 3.80 (0.72) | 3.52 (0.86) | 0.412 | 0.379 | 0.121 |
| Patients request it | 1.88 (0.90) | 1.94 (0.91) | 2.23 (1.08) | 0.726 | 0.049 | 0.234 |
| Minimize the risk of litigation from missing a clinically important finding | 2.21 (0.97) | 2.29 (1.10) | 2.47 (1.12) | 0.881 | 0.153 | 0.384 |
| Consultant would want or request a CT scan prior to consultation | 2.79 (1.20) | 2.69 (1.23) | 3.85 (1.06) | 0.603 | <0.001 | <0.001 |
| EM, emergency medicine; GI, gastroenterology; Surg, surgeons | | | | | | |

**A**

*
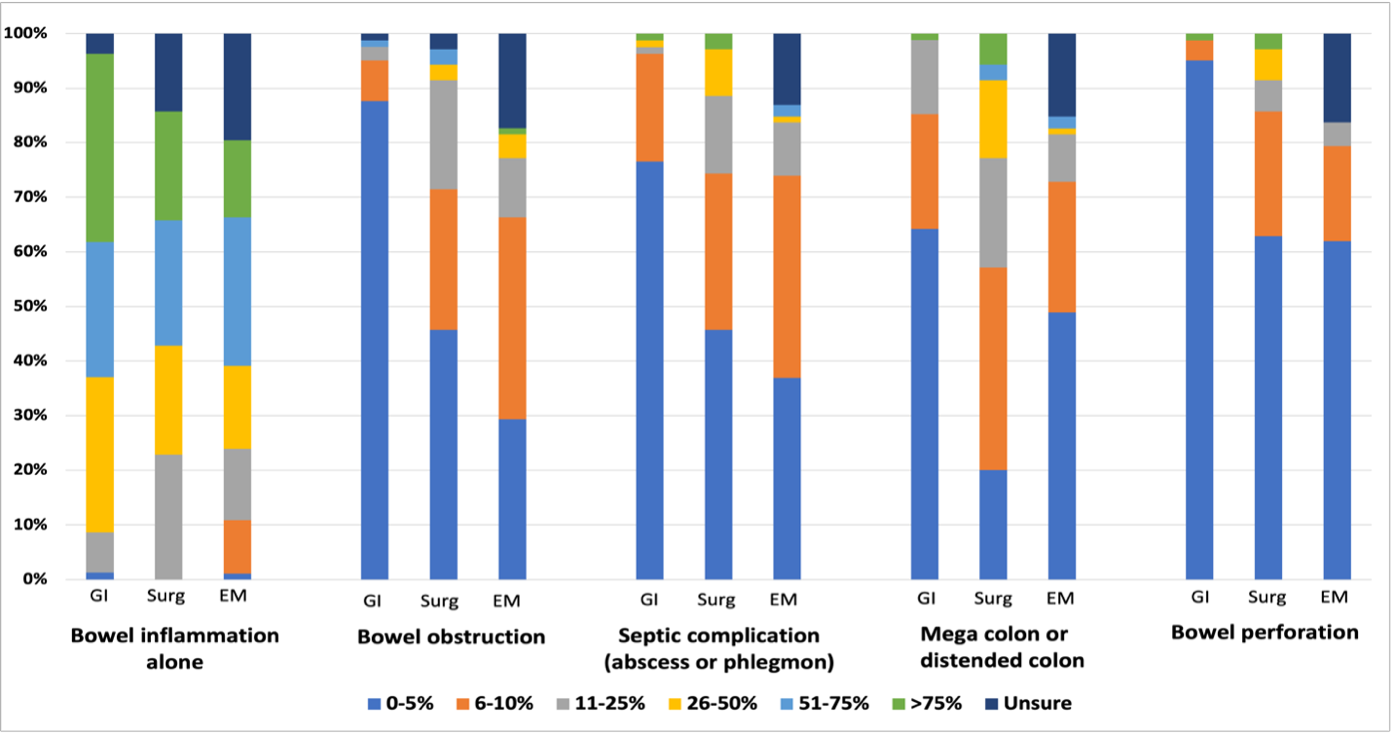
*

**B**

*
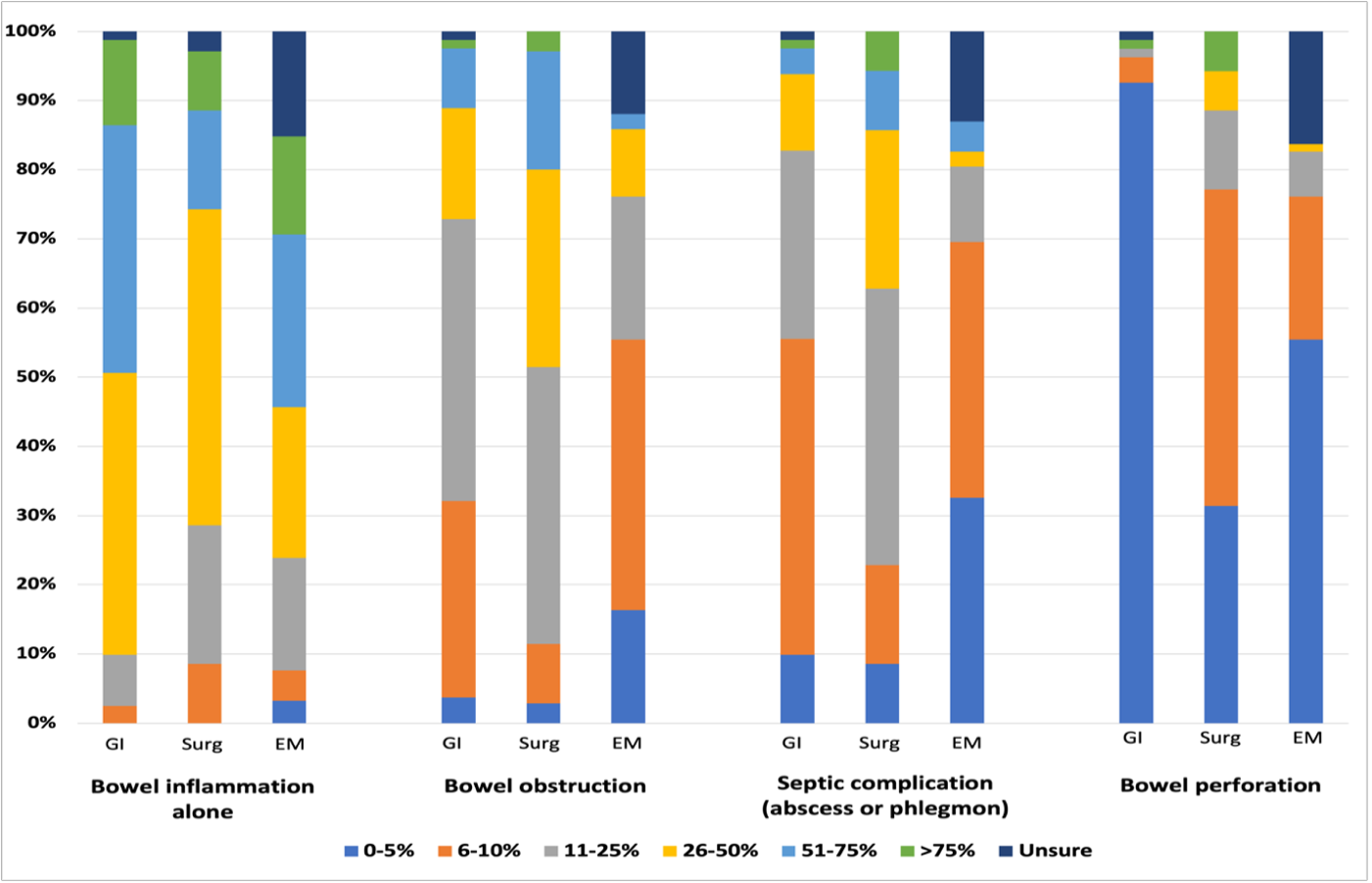
*

**Supplementary Figure 1.** Perceived proportion of specific findings on APCT imaging for patients with a) ulcerative colitis and b) Crohn’s disease in the emergency department stratified by physician speciality: gastroenterology (GI), surgery (Surg), and emergency medicine (EM)

**A**


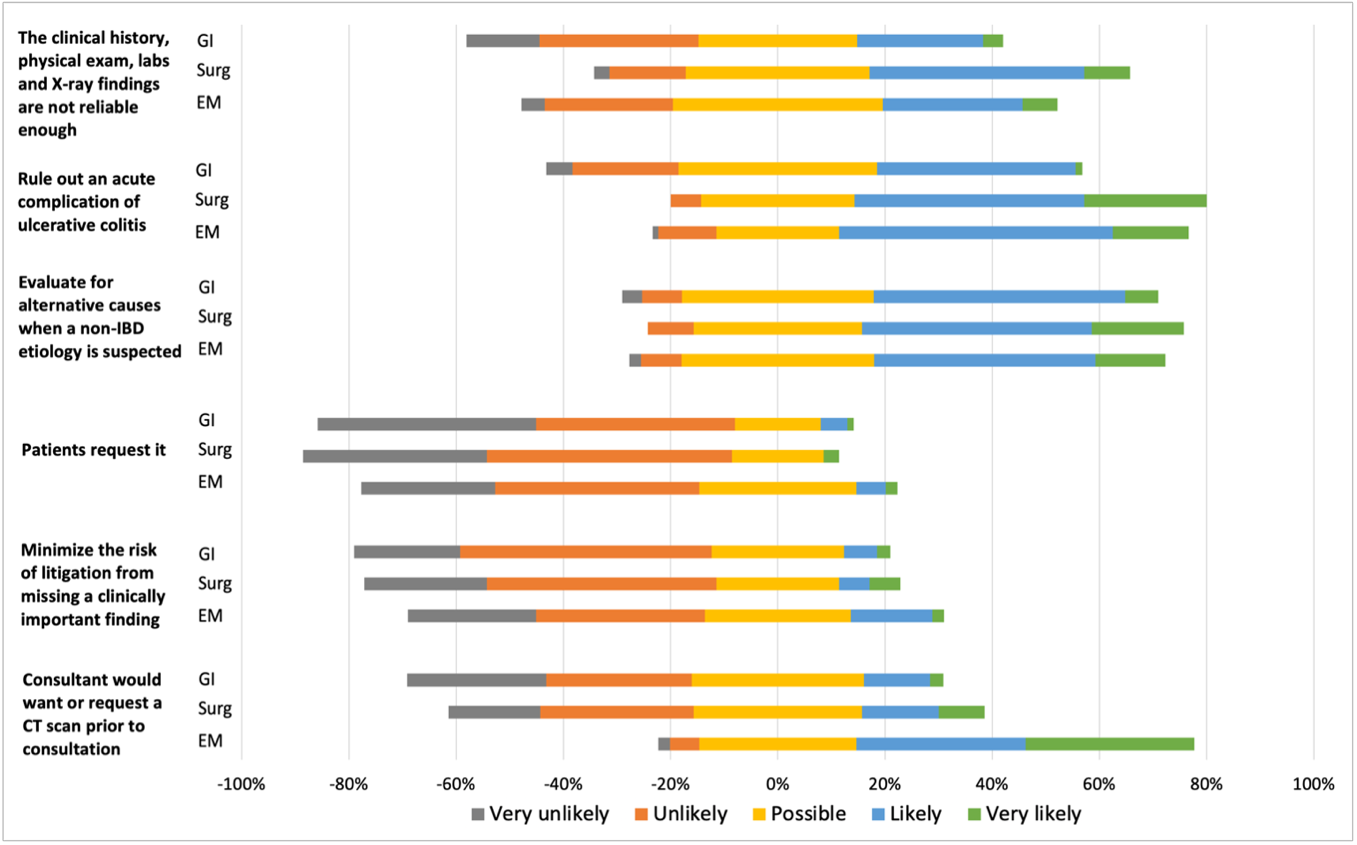


**B**

**
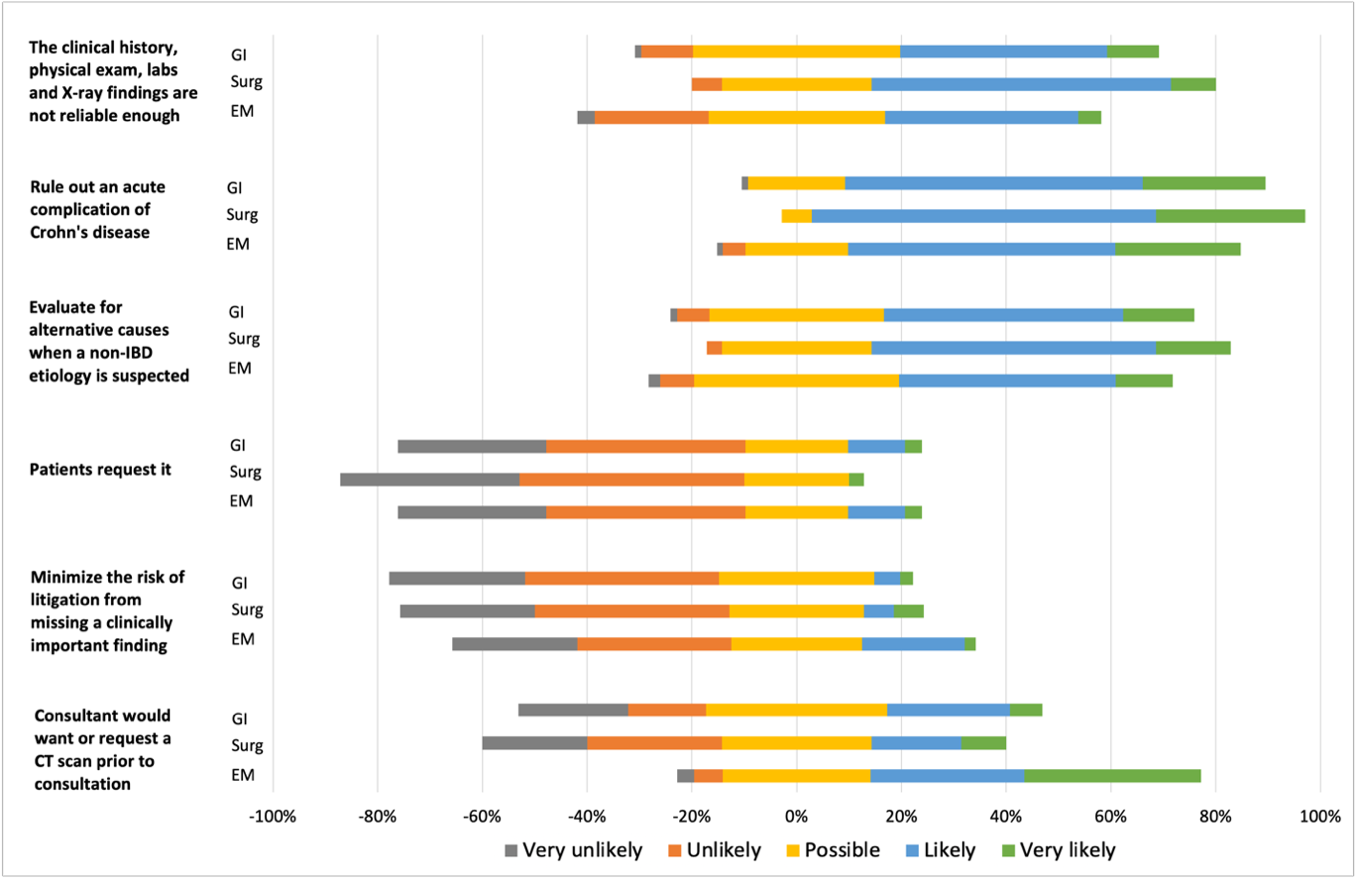
**

**Supplementary Figure 2.** Factors influencing the decision to perform APCT imaging for patients with a) ulcerative colitis and b) Crohn’s disease stratified by physician specialty: gastroenterology (GI), surgery (Surg) and emergency medicine (EM)
