# Supplementary figures and images for: Perceived value of computed tomography imaging for patients with inflammatory bowel disease in the emergency department: a Canadian survey
Source: J Can Assoc Gastroenterol. 2024 Feb 16;7(3):261–8. doi: 10.1093/jcag/gwae001 (PMC11149658; doi:10.1093/jcag/gwae001)

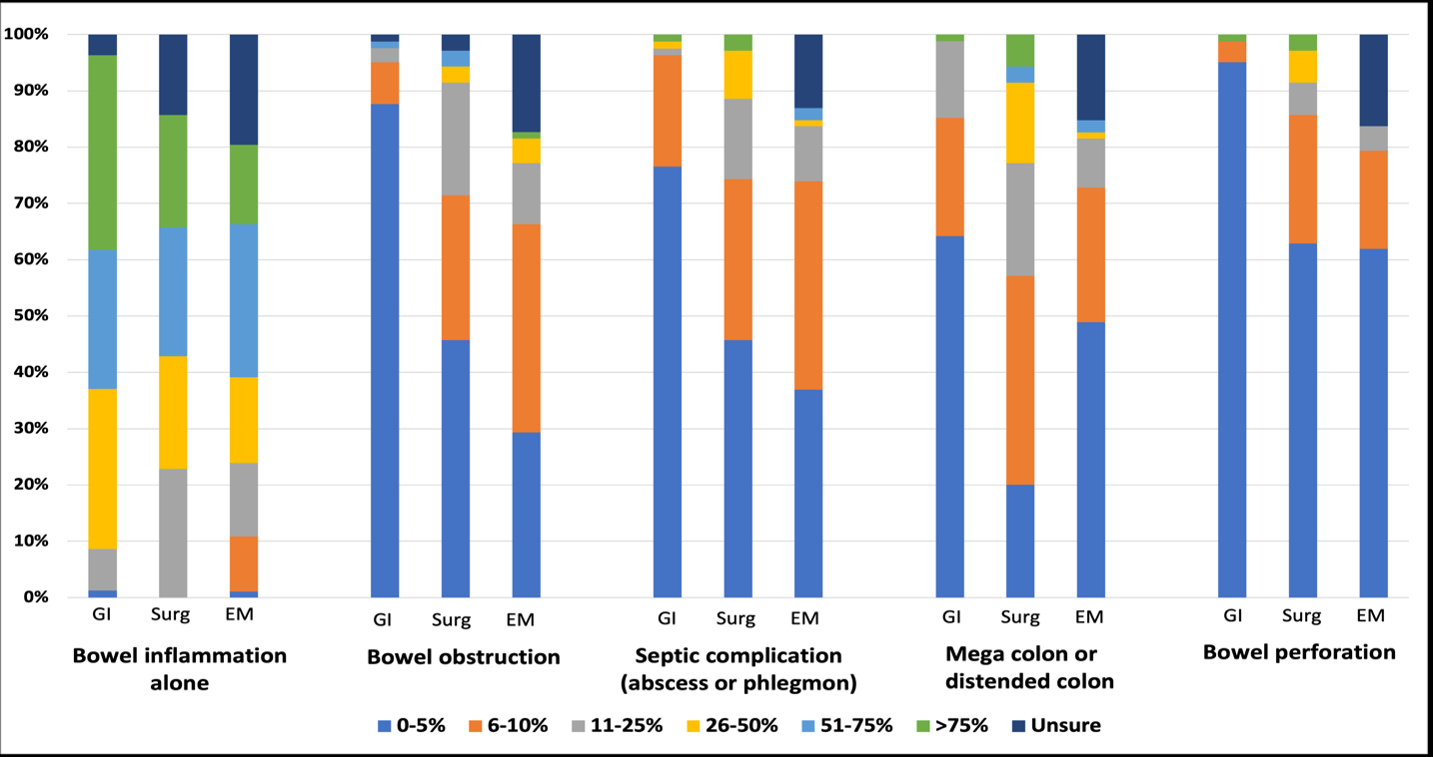

Supplement: gwae001_suppl_Supplementary_Tables_1-4_Figures_1-2 [file gwae001_suppl_supplementary_tables_1-4_figures_1-2.zip › gwae001/Supplimental Figure 1a.tiff]

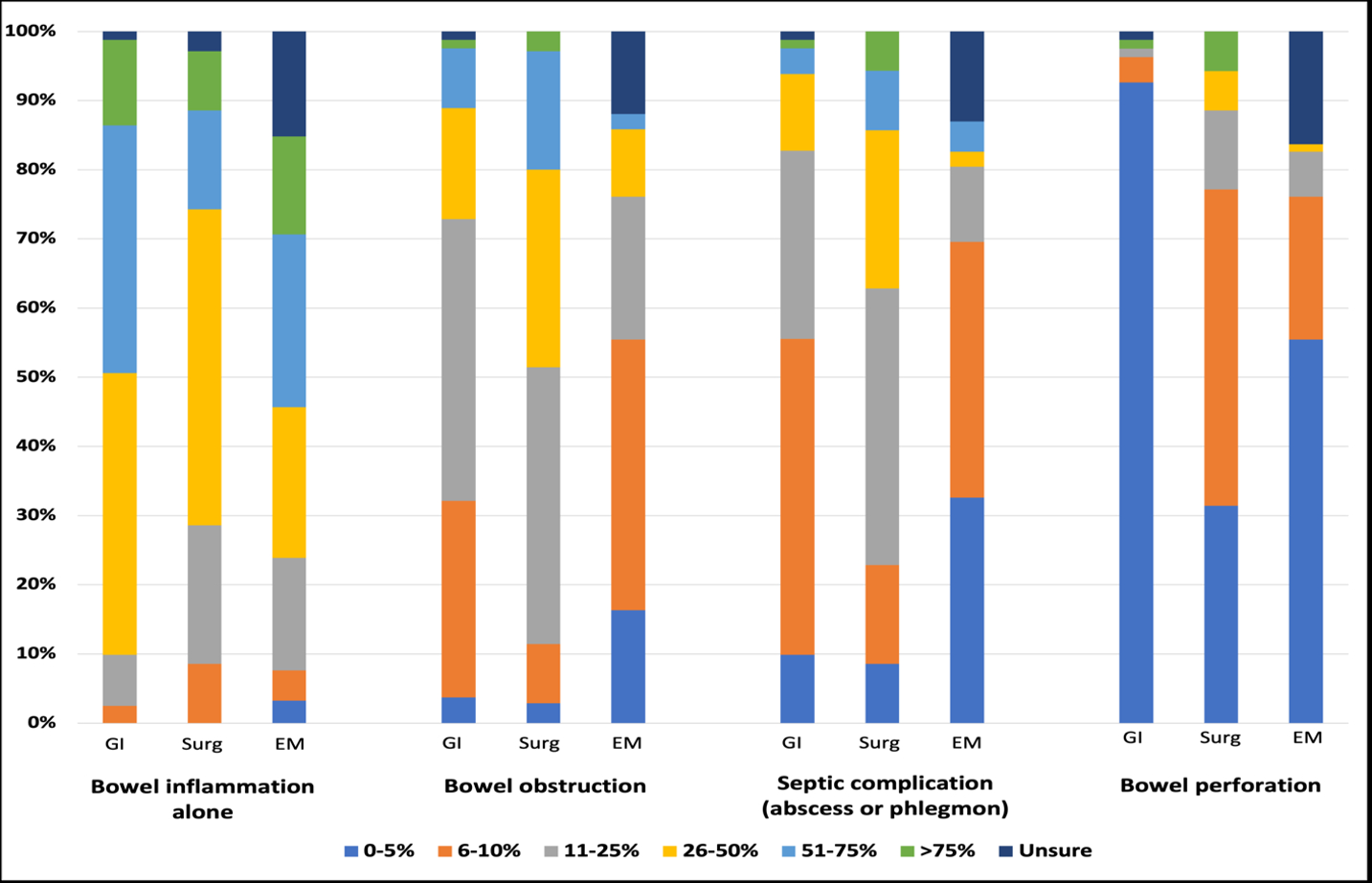

Supplement: gwae001_suppl_Supplementary_Tables_1-4_Figures_1-2 [file gwae001_suppl_supplementary_tables_1-4_figures_1-2.zip › gwae001/Supplimental Figure 1b.tiff]

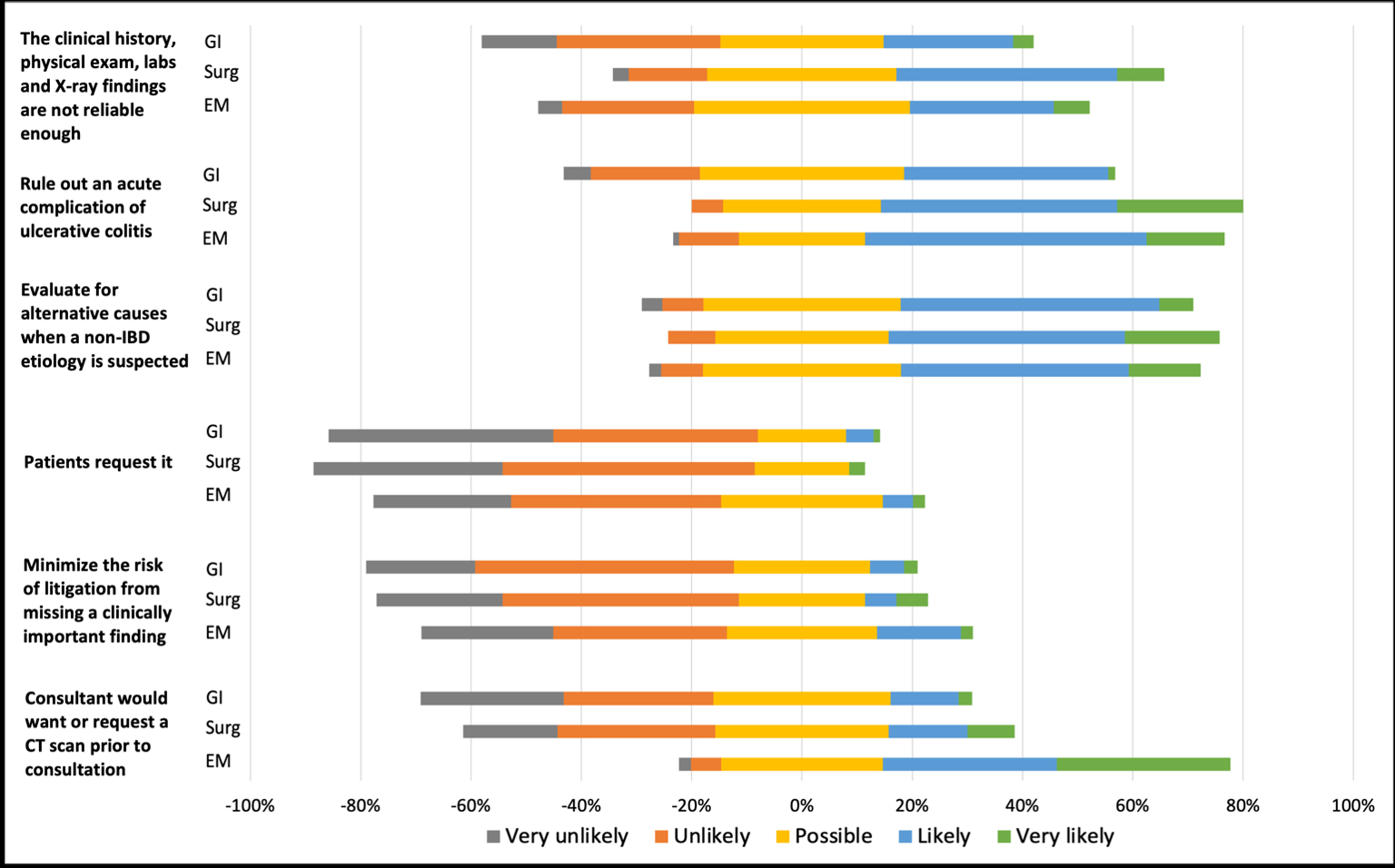

Supplement: gwae001_suppl_Supplementary_Tables_1-4_Figures_1-2 [file gwae001_suppl_supplementary_tables_1-4_figures_1-2.zip › gwae001/Supplimental Figure 2a.tiff]

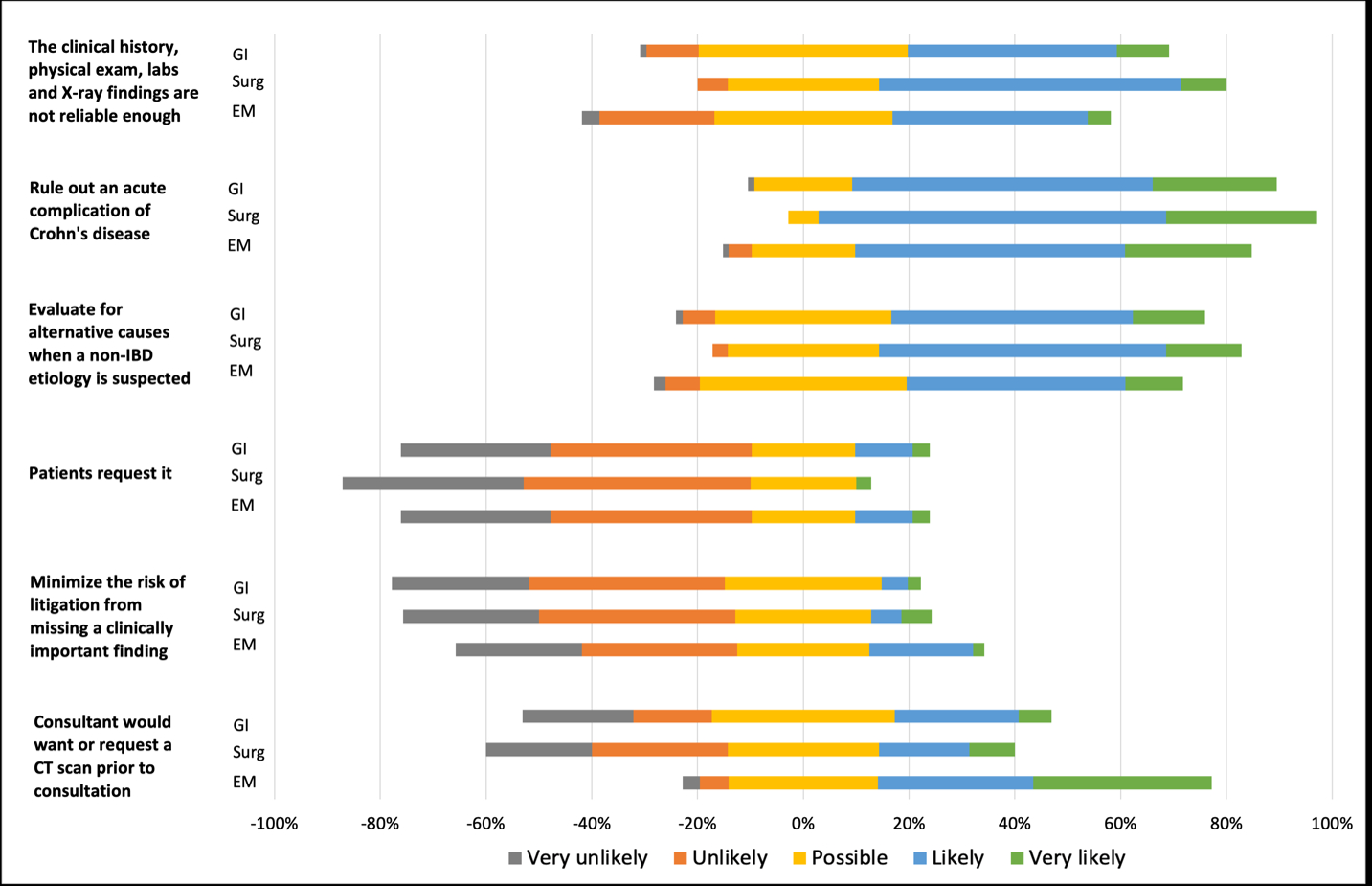

Supplement: gwae001_suppl_Supplementary_Tables_1-4_Figures_1-2 [file gwae001_suppl_supplementary_tables_1-4_figures_1-2.zip › gwae001/Supplimental Figure 2b.tiff]
